# Complete Genome Sequence of *Streptomyces albus* SM254, a Potent Antagonist of Bat White-Nose Syndrome Pathogen *Pseudogymnoascus destructans*

**DOI:** 10.1128/genomeA.00290-16

**Published:** 2016-04-14

**Authors:** Jonathan P. Badalamenti, Joshua D. Erickson, Christine E. Salomon

**Affiliations:** a BioTechnology Institute, University of Minnesota, Saint Paul, Minnesota, USA; b Center for Drug Design, University of Minnesota, Minneapolis, Minnesota, USA

## Abstract

We sequenced and annotated the complete 7,170,504-bp genome of a novel secondary metabolite-producing *Streptomyces* strain, *Streptomyces albus* SM254, isolated from copper-rich subsurface fluids at ~220-m depth within the Soudan Iron Mine (Soudan, MN, USA).

## GENOME ANNOUNCEMENT

White-nose syndrome (WNS) is a devastating disease caused by the psychrophilic fungus *Pseudogymnoascus destructans* which affects bats in the United States and Canada (1). One approach toward disease treatment or prevention is the development of microbial biological control agents for application on or near bats and roost areas (2, 3). We isolated bacteria and fungi from bat swabs, roosts, and other subterranean surfaces near hibernacula areas and screened for antifungal activities in direct competition assays. One *Streptomyces* isolate obtained from high copper sediments in the Soudan Iron Mine exhibited potent antagonistic activity against *P. destructans*. We initiated studies of the genome of *S. albus* SM254 to identify the potential biosynthetic pathways responsible for producing antifungal metabolites.

Sediments were collected from a shallow pool on level 10 of the Soudan Mine (~220-m depth). Samples were diluted in artificial seawater (ASW), vortexed, and plated onto ISP2 media made with ASW and 50 µg/mL cycloheximide and incubated at 25°C for 6 weeks. Genomic DNA for sequencing was obtained using a MoBio Ultrapure Microbial DNA isolation kit.

Reads from 11 PacBio single-molecule real-time (SMRT) cells (P6-C4 chemistry, *N*_50_ = 7,049 bp) were assembled with HGAP v3 at 262× coverage to yield a single linear chromosome, which was polished to quality value (QV) > 50 with successive passes through Quiver (4). The assembly was then further polished using Pilon v1.11 (5) with 80-fold coverage of quality-trimmed (Trimmomatic v0.33) 2 × 250-bp Illumina reads to correct 205 remaining indels, nearly all of which occurred within G or C homopolymer regions. The 7,170,504-bp genome (73.34% G+C) was annotated with Prokka v1.11 (6). Suspect open reading frames (ORFs) which could potentially result from high G+C content were identified by NPACT (7) and manually corrected in all cases where the NPACT-predicted ORF had >90% BLAST identity to other *Streptomyces* proteins. Potential frameshifts were identified using the online submission check tool of the NCBI database (http://www.ncbi.nlm.nih.gov/genomes/frameshifts/frameshifts.cgi), and pseudogenes were called in cases where at least 10× Illumina coverage unambiguously confirmed a predicted frameshift. Finally, custom Python scripts (http://github.com/jbadomics/genbank_submit) were used to assign protein IDs and to bring the curated annotation into compliance with NCBI submission guidelines.

*Streptomyces albus* SM254 shares 99.11% two-way average nucleotide identity with *S. albus* J1074 (http://enve-omics.ce.gatech.edu/ani/), indicating that *Streptomyces albus* SM254 represents a novel strain of *S. albus*. Strain SM254 encodes 6,180 protein-coding genes of which 1,150 have no predicted function, 65 tRNAs, 21 rRNAs, and 16 pseudogenes including tRNA(Ile)-lysidine synthase (TilS), suggesting that this isolate may lack the ability to correctly translate its 1,383 genes which contain at least one AUA codon (8). Like other *Streptomyces* spp., the *S. albus* SM254 genome is replete with biosynthetic genes for secondary metabolites (antiSMASH v3), including terpene, lantipeptide, bacteriocin, nonribosomal peptide synthetase, and polyketide synthase gene clusters.

### Nucleotide sequence accession numbers.

Sequences have been deposited in GenBank under accession number CP014485. Raw Illumina and PacBio reads, as well as base modification data, have been deposited to the NCBI Sequence Read Archive under BioProject PRJNA295319.
